# Coping Behavior of International Late Adolescent Students in Selected Australian Educational Institutions

**DOI:** 10.5539/gjhs.v6n1p76

**Published:** 2013-10-15

**Authors:** Masitah Shahrill, Lawrence Mundia

**Affiliations:** 1 Sultan Hassanal Bolkiah Institute of Education, Universiti Brunei Darussalam, Bandar Seri Begawan, Brunei Darussalam

**Keywords:** coping strategies, coping styles, late adolescents, international students, distress, counseling

## Abstract

Using the Adolescent Coping Scale, ACS (Frydenberg & Lewis, 1993) we surveyed 45 randomly selected foreign adolescents in Australian schools. The coping strategies used most by the participants were: focus on solving the problem; seeking relaxing diversions; focusing on the positive; seeking social support; worry; seeking to belong; investing in close friends; wishful thinking; and keep to self (Table 4). With regard to coping styles, the most widely used was the productive coping followed by non-productive coping while the least used style was reference to others (Table 4). In terms of both genders the four coping strategies used most often were: work hard to achieve; seeking relaxing diversions; focus on solving the problem; and focus on the positive (Table 5). The most noticeable gender difference was the use of the physical recreation coping strategy in which male students engaged more (Fig 1). The usage of four coping strategies (solving problem; work hard; focus on positive; and social support) was higher for students who have been away from family more than once as compared to those who have been away once only while the usage of seeking relaxing diversions was higher for the first timers (Table 6). No significant differences were obtained on the sample’s performance on the ACS subscales by gender (Table 7), frequency of leaving own country (Table 8), country of origin (Table 9), and length of stay in Australia (Table 11). However, foundation students scored significantly higher on the reference to others variable than their secondary school peers (Table 10). We recommended counseling for students with high support needs and further large-scale mixed-methods research to gain additional insights.

## 1. Introduction

Each year, Australia’s universities, institutes, colleges and schools have witnessed an increasing number in international student enrollment. According to an *Australian Trade Commission* (2005) website on overseas students who are considering studying in Australia, more than 180,000 students from 140 countries studied at Australian institutions (either in Australia or in overseas campuses) in the year 2000. Almost 70% of these students undertook courses in the higher education and vocational educational sectors. A recent study by Pritchard (2005) showed that Australia’s 39 universities had about 175,000 foreign students as compared to only 15,000 foreign students twenty years ago. Despite the increased number of international students enrolled in Australian institutions, there has not been much research done to explore the international adolescent students’ coping experiences during their sojourn in Australia. In this study, an international adolescent student is defined as someone who falls within the adolescent age group who enters Australia for the purpose of studying in an Australian institution. The adolescence stage is usually divided into phases, early adolescence (early teens) and late adolscence (late teens). The present study deals with non Australian late adolescents in tertiary Australian educational institutions and will also variously be referred to as “international students” or “foreign students” or “overseas students”. Recent research suggests that post secondary school students need to be assessed not only for academic achievement but also for personal development, growth and problems (Mundia, 2012a). This is because students in tertiary or post secondary school institutions tend to have a wide range of personal problems such as depression, anxiety, and stress (Mundia, 2010a), mental health problems (Mundia, 2010b; Mundia, 2012b) as well as poor academic performance due mainly to adverse effects of psychological distress (Mundia, 2011a). Non-assessment of social problems in school-going late adolescents of developing countries is often attributed to the shortage of adequately trained personnel (e.g. school counselors or educational psychologists) to carry out the evaluations (Mundia, 2009; Mundia, 2010c). Another assessment-related problem is the lack of suitable psychometric instruments to use when obtaining assessment data. Major western scales such as the Revised Eysenck Personality Inventory, EPQ-R and the Revised Minnesota Multiphasic Personality, MMPI-R may be used when suitable adaptations are made (Mundia, 2010d; Mundia, 2011b).

### 1.1 Coping in Adolescence and the Theory of Coping

During adolescence, young people are confronted by a series of developmental hurdles and challenges (Frydenberg, 1997). The period of adolescence is particularly a challenging period (and a crucial time) because developmental and environmental changes often occur simultaneously (Ebata & Moos, 1991, 1995; Thuen & Bru, 2004). The factors (or challenges) young people deal with range from the onset of puberty, the increasing need for independence, the influence of parents, differentiating themselves from their family, forming bonds with peers, and societal expectations of achievement to vocational choices and opportunities (McKenzie & Frydenberg, 2004; Richaud de Minzi, 2003).

Subsequently, it is their coping behaviours that will identify how adolescents deal with their stresses. According to Frydenberg (1997, 1999a), coping consists of the responses (thoughts, feelings and actions) that are called upon by an individual to deal with problematic situations (i.e. stresses), encountered in everyday life and in particular circumstances. Further, Mundia (2010e) states that late adolescents in tertiary institutions use a variety of coping strategies that are task-oriented, emotional-oriented and avoidance-oriented. However, they often need counseling or psychotherapy when the presenting problem is severe or profound.

The transactional theory of coping represents one of the major theoretical approaches to stress and coping. This theory was derived from the conceptualisation of the work on stress and coping by Richard Lazarus and his colleagues (Folkman & Lazarus, 1985, 1988; Lazarus, 1991; Lazarus & Launier, 1978). The transactional theory of coping is described as the interaction by an individual with their environment. The theory is based on two theoretical frameworks, namely, the *cognitive phenomenological theory*, also labelled as *cognitive transactional theory* (where the individual reacts to the environment as they perceive it) and the *person-environment interaction model* (where the individual and the environment are in a constant state of action and reaction) (Frydenberg, 1997).

According to Lewis and Frydenberg (2004a), much of the coping research in the child and adolescent arena were mostly based on the transactional theory of coping generated by Folkman and Lazarus. In contrast, Hobfoll’s (1988) *Conservation of Resource (COR) Theory* emerged in 1988. Hobfoll (1988, 2002) defined resources as those objects (or entities), personal characteristics, conditions, or energies that are centrally valued by the individual in their own right (e.g. self-esteem, close attachments, health, and inner peace) or the means for attainment of centrally valued ends (e.g. money, social support, and credit). This resource theory emphasises the role of interacting resources, that is, there is a ‘commerce of resources’ which accounts for the fact that some people manage to adapt despite the circumstances they encounter (Frydenberg & Lewis, 2002; McKenzie & Lewis, 2004). The COR theory assumes the fact that people seek to obtain, retain and protect resources (Hobfoll, 2002). From this model, Hobfoll suggested that stress occurs when there is a loss of resources, when the resources are threatened with loss or lost, or the resources are poorly gained by individuals after substantive resource investment (Frydenberg & Lewis, 2002; Hobfoll, 1988, 2002; McKenzie & Frydenberg, 2004; Schwarzer & Taubert, 2002). Hobfoll’s theory is considered less tested, in the sense that not much research (apart from the most recent study by McKenzie and Frydenberg (2004)) was put forward to practice this theory in order to identify the resources important to young people.

Lazarus argued that there are three processes involved in stress, namely primary appraisal (the process of an individual perceiving a threat to his/her resources or well-being), secondary appraisal (the process of bringing to mind a potential response i.e. he/she possesses the coping resources to manage the threat), and coping (the process of executing that response) (Carver, Scheier & Weintraub, 1989; McKenzie & Frydenberg, 2004). Folkman and Lazarus (1985) stated that the two components of cognitive appraisal (primary and secondary) operate interdependently. For example, if a coping response is found to be less effective than expected, the outcome is to review or re-appraise (tertiary appraisal or reappraisal) the level of threat or what coping response is appropriate. The entire set of processes may cycle repeatedly illustrating the fact whether the strategies are likely to be tried again or rejected from future use according to the effectiveness of the outcome by an individual (Carver *et al.*, 1989; Folkman & Lazarus, 1985; Frydenberg, 1997, Frydenberg & Lewis, 2002; Lewis & Frydenberg, 2004b).

There are two major types of coping strategies; problem-focused coping (aimed at problem solving, or doing something to alter the source of stress, for example, the strategies such as situational control, positive self-instructions and support-seeking) and emotion-focused coping (aimed at reducing or managing the emotional distress that is associated with the situation, for example, the strategies such as minimisation and distraction or recreation) (Carver et al., 1989; Ebata & Moos, 1991; Essau & Trommsdorff, 1996; Folkman, 1997, Hampel & Petermann, 2005; Hashim, 2003; McKenzie & Frydenberg; 2004; Thuen & Bru; 2004). Furthermore, these two types of coping strategies have been widely used in research on coping in childhood and adolescence. However, Compas, Connor-Smith, Saltzman, Thomsen and Wadsworth (2001) argued that criticism concerning these two types is also widespread because “they are overly broad and place many disparate types of coping into these two general categories” (p. 92).

One might argue that there are many factors that contribute to how young people cope. General research in the field of coping (such as the link between coping and well-being) has identified characteristics with more effective coping in adolescence, thus enabling them to cope with stress. The characteristics include temperament, optimism, perceived personal control, familial factors (such as family cohesion, shared values, loving parents, and a relationship with at least one parent figure), flexibility and the availability of social support (Lewis & Frydenberg, 2004a, 2004b). However, in the pursuit of social, emotional and developmental maturity, how a young person copes is determined by peer and familial factors (Frydenberg, 1999b, 2004).

As stated earlier, most of the research on international students was based in the United States (for example; Chen, 1996; Chiu, 1995; Constantine, Anderson, Berkel, Caldwell & Utsey, 2005; Cross, 1995; Nasrin, 2001; Wan, Chapman & Biggs, 1992; Zhai, 2002). However, McKnight and Turner (1995) stated that there have been numerous studies of the particular needs (mainly on linguistic, cultural and personal difficulties in learning in a different language and culture) of international students in Australia. In contrast, the study by Hashim (2003) on international students was based in China. These writers, however, focused primarily towards international students at the university level. Hence, presented next is the literature reviewed on international students with regards to the variety of problems or experiences they encountered and the coping strategies used whilst sojourning in countries other than their own.

### 1.2 International Students’ Experiences

According to Myburgh, Niehaus and Poggenpoel (2002), “studying and living in another country for a period of time enable international learners to explore new cultures and to broaden their life experiences through exposure to these cultures and foreign places”, (p. 107). However, trying to adjust and adapt to a new environment or culture is not an easy task, especially being thousands of kilometres away (or oceans apart) from family and friends. Pennebaker, Colder and Sharp (1990) stated,

Leaving home and moving to a college dormitory around age 18 is a major upheaval for most students. The transition to college involves leaving family and friendship networks. In addition to coping with new living arrangements, most entering freshmen are changing roles within their families and within society in general. Further, most face more difficult courses and greater levels of social and academic competition than ever before. (p. 530)

The statement above may also be taken in the context of international students as well. The transition of international students studying abroad (especially within the adolescent age group) involves leaving family and friendship networks at a very young age. These young international learners face many challenges such as personal, academic and adjustment problems. Nasrin’s (2001) study which investigated international female graduate students’ perceptions of their adjustment experiences has identified three categories of the adjustment problems encountered by international students, namely; academic issues (i.e., language and communication), personal adjustment problems (i.e., homesickness, housing and financial problems), and in social area (i.e., culture shock and discrimination). In fact, Cross (1995) pointed out that factors such as gender, age, marital status, housing arrangements, financial support, language ability, and previous experience in other cultures often predict international student adjustment. Cross (1995) found that East Asian students (studying in the U.S.) whose self-views and coping strategies were consistent with the amin culture’s norms and ideals experienced lower levels of perceived stress (stressors such as language difficulties, new norms and social customs, and challenges to one’s self views and beliefs) than did other East Asian students.

The phenomenon of culture shock has been identified in several studies as one of the many challenges faced by international students (see Chen, 1996; Hashim, 2003; McKnight & Turner, 1995; Nasrin, 2001; Ryan & Twibell, 2000; Zhai, 2002). According to Winkelman (1994), culture shock is normal in a foreign culture environment and by preparing for problems and using the resources to minimise culture shock, will promote coping and adjustment. Funaki (1995) (as cited by Chen, 1996) stated “international students use coping mechanisms rather than defense mechanisms to deal with the problem of culture shock” (p. 17). In Chen’s (1996) study of understanding how five international graduate students perceive their experience as foreign students found that ‘learning by reflective thinking’ as the most significant process in dealing with the problems of culture shock. The other main findings in Chen’s study was ‘the feeling of loneliness’ experienced by international students. It was revealed that these students had no time to make new friends in the new country due to heavy demands of academic work and the language limitation.

In terms of international students’ help-seeking behaviours, Zhai (2002) reported 7 out of the 10 international participants (in Zhai’s study) preferred their friends or family as the most favoured resource to seek help (regarding personal problems and issues). Furthermore, Zhai (2002) stated that the students “… felt very connected with their fellow international students and very isolated from U.S. students”, (p. 8). In a study by Constantine et al. (2005), they examined the cultural adjustment experiences of African international college students studying in U.S. where the results indicated that the Kenyan, Nigerian and Ghanaian students used several coping strategies to deal with stressful situations. Similar to Chen’s (1996) results, the participants in Constantine et al.’s (2005) also reported, “that they felt lonely and isolated from others” (p. 62). In terms of ‘strategies for coping with cultural adjustment problems’ and ‘openness to seeking counseling to address cultural adjustment problems’, Constantine *et al*. (2005) offered descriptions of these two categories by stating,

Participants typically reported that they sought social support from both family members and friends to cope with problems related to adjusting to U.S. culture… With regard to their cultural adjustment problems, participants also occasionally indicated that they (a) kept problems to themselves so as not to trouble or burden others (i.e. forbearance), (b) engaged in physical activities such as exercise, and (c) slept to avoid problems… Participants typically reported that they were not open to the idea of seeking counseling to address their cultural adjustment difficulties. (p. 62)

Hashim’s (2002) study however, investigated the stress and coping behaviours of 76 students from Western countries (North America, Europe, Australia and New Zealand) and 83 students from Africa, attending 11 universities in three cities in China (namely; Beijing, Shanghai and Guangzhou). The results indicated Western students demonstrated the highest ability to cope with stress when compared to the African students. In addition, both African and Western students showed low ability in resourcefulness (i.e., weak ability to find solutions to uncommon problems and do not seem to know where to turn for help, information, or support) while trying to cope with stress of studying abroad in China.

Growing concerns have been highlighted in these studies, however, mostly in the United States, with regards to coping with cultural differences and the academic demands which Zhai (2002) identified as the important adjustment issues for international students. Most of the articles cited here were mainly researches on international students at university level and none of the studies dealt specifically at high school or college level (that focuses on the adolescent age group). Since there is insufficient research on coping of international adolescent students studying in Australia, this study was conducted with the hope of identifying the coping behaviours of international adolescent students while attending the Australian education system.

### 1.3 Objectives of the Study

The aim of this study is to investigate how international adolescent students cope whilst being away from their family and friends back home. Specifically, the study sought to compare the coping strategies of the international adolescent students by gender, age-group, educational level, country of origin and whether they were away from home for the first time or not.

## 2. Method

The study used the field survey approach to investigate the problem. This research strategy differs from the telephone, postal, and online survey techniques in that the researcher has to go into the fields (relevant educational institutions in the present study) to collect the data. The rationale and justification for employing this research strategy was two-fold. First, we wanted to involve as many foreign students in the study as possible. Second, it was then possible to give on-the-spot assistance to respondents who needed help to complete the data collection instruments correctly thereby increasing the number of usable returns.

### 2.1 Sample

A total of 45 questionnaires (out of the 62 distributed) were collected giving a response rate of 73%. The composition and demographic characteristics of the sample were as presented in Table 1. Participant students reported that they were in Australia between one to fifteen months. By educational level of the 45 respondents, two female students were in Year 10, five students in Year 11 (2 female and 3 male), five students in Year 12 (4 female and 1 male) and 33 students (16 female and 17 male) enrolled in a foundation course. A foundation course is a seven to eight months intensive course that served as an alternative course for gaining entry into university. Only one of the students attended one of the Melbourne University colleges whereas the rest were from a college in the city.

**Table 1:** Demographic information (N = 45)

Variable	Group	Frequency	Percentage
Gender	Females	24	53.300
	Males	21	46.700
Program	Secondary (Years 10-12)	14	31.100
	Foundation (Taylor & Trinity)	31	68.900
Country	Hong Kong	9	20.000
	Singapore	9	20.000
	Indonesia	8	17.800
	Malaysia	11	24.400
	Others*	8	17.800
Age		Mean	SD
	All	17.733	1.213
	Females	17.791	1.350
	Males	17.666	1.213

* China (3); Korea (2); Nepal (1); Bangladesh (1); Zimbabwe (1)

### 2.2 Instruments

The instrument used in this study was the long form Adolescent Coping Scale, ACS, designed by Frydenberg and Lewis (1993a) to measure coping. This coping questionnaire is an 80-item Likert-type instrument developed to identify the coping strategies and styles used to deal with stress during adolescence. The instrument was developed and normed Australia (Frydenberg, 1997, 2004; Frydenberg & Lewis, 2000, 2004). The eighteen coping strategies have been grouped into three basic coping styles, namely, problem solving (also known as productive coping style), relations with others (or reference to others), and non-productive coping. The coping styles represent both functional strategies (e.g. direct attempts to confront the problem with or without reference to others) and dysfunctional strategies (e.g. the use of non-productive techniques such as avoidance, ignoring, self-blame, and wishful thinking) aspects of coping (Frydenberg, 1997; Frydenberg & Lewis, 1993b, 2000, 2004; Lewis & Frydenberg, 2004a; McKenzie & Frydenberg, 2004; Richaud de Minzi, 2003). Attached to the ACS was a page requiring the respondents to fill in their background information such as their age, country of origin, the date or month of their first arrival to Australia and so on. The reliability and validity of the data collection scales are presented in Table 2 and Table 3 respectively.

**Table 2:** Reliability of the data collection instruments (N = 45)

Scale	Items	Mean	SE Mean	SD	Alpha
Productive Coping Subscale (PCS)	35	117.688	2.197	14.739	0.842
Reference to Others Subscale (ROS)	17	46.044	1.540	10.335	0.828
Non-productive Coping Subscale (NCS)	37	103.066	2.929	19.651	0.910
Overall ACS Scale* (OACS)	89	234.088	4.096	27.478	0.886

* ACS = Adolescent Coping Scale (Frydenberg & Lewis, 1993)

**Table 3:** Convergence and discriminant validity of the data collection instruments (N = 45)

Scale†	1	2	3
1. PCS	1		
2. ROS	0.569**	1	
3. NCS	0.042	0.243	1
4. OACS	0.580**	0.711**	0.779**

† See Table 2 for full scale names

** p < .01 (2-tailed)

### 2.3 Data Analysis

The quantitative data were analyzed by both descriptive statistics (frequencies, percentages, mean and standard deviation) and inferential statistics (t-tests for independent samples incorporating ANCOVA F, Pearson’s correlation, and One-Way ANOVA). The rationale and justification for using these techniques is two-fold. First, the procedures were deemed to be appropriate for addressing the research objectives. Second, the data were obtained from a random sample and there was no evidence regarding violation of the statistical assumptions.

### 2.4 Procedures

The study was carried out at a student accommodation facility in one city of Australia. Almost all of the residents were international students studying at several Australian educational institutions in the city. The majority of the residents fell within the adolescent age group. In total, 62 questionnaires were distributed and students were told to return the questionnaire within four days. At the start of distributing the questionnaires, students were told that their participation in the study was purely voluntary. Other ethical conditions for obtaining data from human participants were followed as recommended by the Helsinki Declaration.

## 3. Results

A majority of the students reported that their main concern was ‘school’ (since 84% were enrolled in the foundation course preparing them to gain entry or accessto their desired university. Table 1 contains the sample’s descriptive statistics based on the coping strategies and styles. To facilitate comparison of usage of the various strategies, the number of items in each scale and the average item mean per scale are also provided and ranked according to their usage.

### 3.1 Coping Strategies and Styles for All Participants

The coping strategies used most often to manage their concerns were Focus on Solving the Problem, Focusing on the Positive, Seeking Social Support, Worry, Seeking to Belong, Investing in Close Friends, Wishful Thinking, and Keep to Self. Only two strategies were used ‘very little’, these were Social Action and Not Coping. In general, these international students were coping well (as indicated by the low average item mean scores. The results for the coping styles (in Table 1) indicated that the most common used style was the Productive Coping, followed by Non-Productive Coping and the least used style was Reference to Others.

**Table 4:** Sample relative usage of coping strategies and styles arranged by average item mean (n = 45)

Coping strategies	No. of items	Mean	SD	Average item mean
17. Relaxing diversions	3	11.13	1.98	3.71
3. Work hard	5	17.98	3.34	3.60
2. Solving problem	5	17.93	3.02	3.59
15. Focus on positive	4	13.82	2.74	3.46
1. Social support	5	16.73	3.85	3.35
4. Worry	5	16.09	3.57	3.22
6. Belong	5	16.00	3.55	3.20
5. Friends	5	15.76	4.68	3.15
7. Wishful thinking	5	15.73	4.50	3.15
13. Keep self	4	12.09	2.78	3.02
12. Self-blame	4	11.33	3.25	2.83
18. Physical recreation	3	8.27	2.73	2.76
14. Spiritual support	4	11.00	5.01	2.75
9. Tension Reduction	5	11.69	3.94	2.34
11. Ignore the problem	4	9.18	2.82	2.29
16. Professional help	4	9.16	4.01	2.29
10. Social action	4	9.11	2.92	2.28
8. Not coping	5	10.96	3.80	2.19

Coping styles				
Non-productive	37	103.07	19.65	2.79
Productive	35	117.62	14.68	3.36
Reference to others	17	46.00	10.34	2.71

### 3.2 Coping Strategies and Styles by Gender

**Figure 1:**
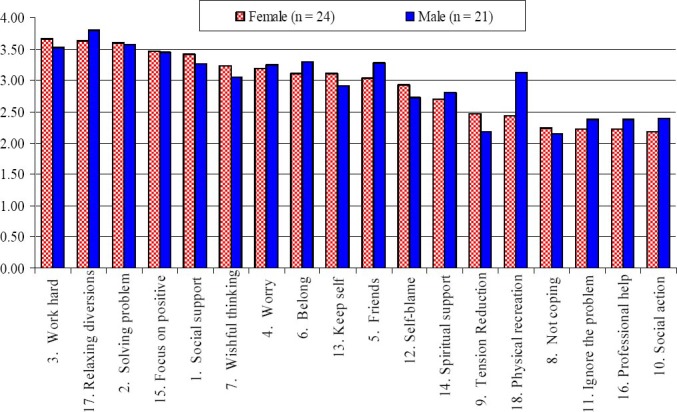
Comparison of the coping strategies by gender

Table 5 consists of the means and standard deviations for male and female students’ usage of the 18 coping strategies followed by the three coping styles. A visual comparison between female and male students’ usage of the 18 coping strategies is provided in Figure 1 (note also that the graphs for male students have been arranged following the rank of female students’ usage of the strategies).

**Table 5:** Gender comparisons of means and standard deviations of international adolescent students’ usage of coping strategies and styles (N = 45)

Coping strategies	Female (n = 24)			Male (n = 21)		
	Mean*		SD	Mean*		SD
1. Social support	(5)	3.42	0.93	(7)*	3.27	0.55
2. Solving problem	(3)	3.60	0.68	(2)	3.57	0.52
3. Work hard	(1)	3.66	0.68	(3)	3.52	0.66
4. Worry	(7)	3.19	0.83	(8)	3.25	0.57
5. Friends	(10)	3.04	1.05	(6)	3.28	0.79
6. Belong	(8)	3.12	0.81	(5)	3.30	0.59
7. Wishful thinking	(6)	3.23	0.90	(10)	3.05	0.91
8. Not coping	(15)	2.23	0.68	(18)	2.14	0.86
9. Tension Reduction	(13)	2.48	0.84	(17)	2.18	0.71
10. Social action	(18)	2.18	0.81	(14)	2.39	0.63
11. Ignore the problem	(16)	2.23	0.69	(15)	2.37	0.73
12. Self-blame	(11)	2.93	0.84	(13)	2.73	0.79
13. Keep self	(9)	3.11	0.72	(11)	2.92	0.67
14. Spiritual support	(12)	2.70	1.38	(12)	2.81	1.12
15. Focus on positive	(4)	3.46	0.74	(4)	3.45	0.64
16. Professional help	(17)	2.22	1.09	(16)	2.37	0.91
17. Relaxing diversions	(2)	3.64	0.71	(1)	3.79	0.61
18. Physical recreation	(18)	2.43	0.80	(9)	3.13	0.90

Coping styles				
Non-productive	(2)	2.82	0.58	(2)	2.75	0.48
Productive	(1)	3.32	0.41	(1)	3.41	0.43
Reference to others	(3)	2.67	0.69	(3)	2.74	0.51

* Values in parentheses indicate the rank of strategy’s usage

There were four coping strategies used most often by female and male students. These were Work Hard to Achieve, Seeking Relaxing Diversions, Focus on Solving the Problem, and Focus on the Positive. As shown in Figure 1, the most noticeable gender difference is the use of the Physical Recreation coping strategy. Male students engaged more in Physical Recreation (mean = 3.13) than the female students (mean = 2.43). Overall, the mean usage of the other coping strategies by female international adolescent students is at par with their male counterparts.

With regards to the coping styles, the results were fairly consistent for both the male and female students. Productive coping was the style used most by both samples, although, male students used this style more than the female students. In contrast to the Non-productive coping style, female students used it more than the male students. Reference to others was the least used coping style for the students.

### 3.3 Coping Strategies and Styles by Transition Experiences

Of the 45 respondents in the present study, 51% (13 females and 10 males) reported that it was their first time being away from family whereas 49% (11 females and 11 males) indicated that they have been away more than once(mainly to boarding school and school excursions to another country). Most of the respondents said that the decision to study in Australia was mainly made by them and primarily supported by parents, siblings, relatives, and friends.

**Figure 2:**
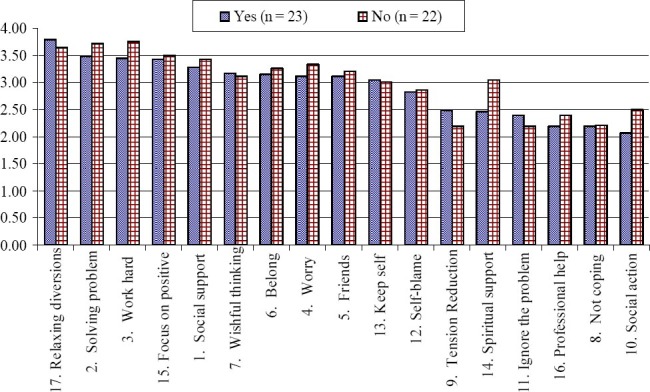
Comparison of the coping strategies by transition experiences

**Figure 3:**
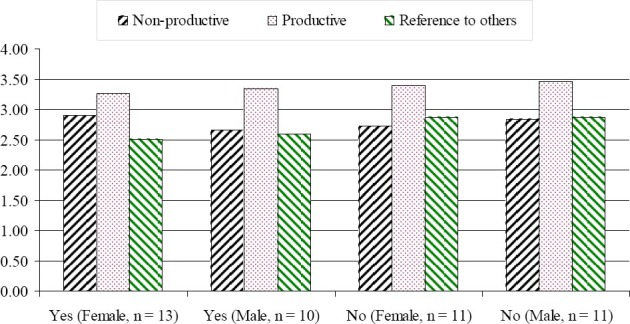
Comparison of the coping styles by transition experiences and gender

The means and standard deviations of the coping strategies and styles presented in Table 6. Figure 2 and Figure 3 represent the visual effect of the results in Table 6 (arranged according to the rank of the “Yes” responses). The mean usage of the four coping strategies (Solving Problem, Work Hard, Focus on Positive and Social Support) was higher for students who have been away from family more than once as compared to students who haven’t been away from family. In contrast, the usage of Seeking Relaxing Diversions was higher for the first timers. As shown in Figure 2, the most noticeable transitional level difference in the mean was Seeking Spiritual Support and Social Action (where the ‘No’ students used these two coping strategies more frequently than the ‘Yes’ students). Overall, the ‘No’ international adolescent students indicated that they used 12 coping strategies more frequently than the ‘Yes’ international adolescent students.

**Table 6:** Means and standard deviations for ‘first-time away’ (Yes) and ‘haven’t-been away’ (No) international adolescent students (N = 45)

Coping strategies	Yes (n = 23)			No (n = 22)		
	Mean*		SD	Mean*		SD
1. Social support	(5)	3.28	0.82	(5)	3.42	0.72
2. Solving problem	(2)	3.47	0.61	(2)	3.71	0.59
3. Work hard	(3)	3.44	0.75	(1)	3.75	0.54
4. Worry	(8)	3.11	0.77	(6)	3.33	0.65
5. Friends	(9)	3.10	0.98	(8)	3.20	0.90
6. Belong	(7)	3.15	0.78	(7)	3.25	0.64
7. Wishful thinking	(6)	3.17	0.91	(9)	3.12	0.91
8. Not coping	(17)	2.18	0.86	(16)	2.20	0.66
9. Tension Reduction	(13)	2.48	0.85	(18)	2.19	0.70
10. Social action	(18)	2.07	0.63	(14)	2.50	0.78
11. Ignore the problem	(15)	2.39	0.80	(17)	2.19	0.59
12. Self-blame	(11)	2.82	0.95	(12)	2.85	0.67
13. Keep self	(10)	3.03	0.82	(11)	3.01	0.56
14. Spiritual support	(14)	2.47	1.16	(10)	3.05	1.31
15. Focus on positive	(4)	3.42	0.74	(4)	3.49	0.63
16. Professional help	(16)	2.18	0.91	(15)	2.40	1.10
17. Relaxing diversions	(1)	3.78	0.66	(3)	3.64	0.67
18. Physical recreation	(12)	2.71	0.96	(13)	2.80	0.88

Coping styles						
Non-productive		2.80	0.57		2.78	0.50
Productive		3.30	0.45		3.43	0.38
Reference to others		2.54	0.50		2.87	0.68

* Values in parentheses indicate the rank of strategy’s usage

### 3.4 Participants’ Performance on the ACS Subscales by Gender

As shown in Table 7, no significant gender differences were obtained on the four subscales.

**Table 7:** ACS subscale means, standard deviations and T-values by gender (N = 45)

Scale†	Females (n = 24)		Males (n = 21)		ANCOVA	T	P
	Mean	SD	Mean	SD	F	(*df* = 43)	(2-tailed)
PCS	116.333	14.648	119.238	15.049	0.021	-0.655	0.516
ROS	45.541	11.758	46.619	8.680	1.860	-0.345	0.732
NCS	104.333	21.363	101.619	17.906	1.037	0.458	0.649
OACS	233.291	30.929	235.000	23.651	1.529	-0.206	0.838

† See Table 2 for full scale names

### 3.5 Participants’ Performance on the ACS Subscales by Number of Times Away From Home Country (N = 45)

Again, no significant differences were obtained by transition times on all the subscales (see Table 8). However, the difference on the ROS subscale nearly reached the lowest significance level.

**Table 8:** Means, standard deviations and T-values for number of times away from family (N = 45)

Scale†	First time away from family (n = 23)		Not first time away from family (n = 22)		ANCOVA	T	P
	Mean	SD	Mean	SD	F	(*df* = 43)	(2-tailed)
PCS	115.391	15.876	120.090	13.391	0.785	-1.071	0.290
ROS	43.260	8.497	48.954	11.437	2.150	-1.901	0.064
NCS	103.434	21.088	102.681	18.519	0.331	0.127	0.900
OACS	230.087	25.964	238.272	28.891	2.118	-0.999	0.323

† Full scale names are in Table 2

### 3.6 Participants’ Performance on the ACS Subscales by Country of Origin (N = 45)

No significant differences were detected by participants’ country of origin (Table 9).

**Table 9:** Means, standard deviations and F-values by country (N = 45)

Scale†	Hong Kong (n=9)		Singapore (n = 9)		Indonesia (n = 8)		Malaysia (n = 11)		Others^a^ (n = 8)		F	P
	Mean	SD	Mean	SD	Mean	SD	Mean	SD	Mean	SD	(*df*=4;44)	(2-tailed)
PCS	120.000	17.881	120.444	11.424	117.125	11.482	115.909	17.529	115.000	15.811	0.226	0.922
ROS	51.111	9.980	40.888	6.071	46.875	10.273	44.636	12.233	47.250	11.106	1.215	0.320
NCS	110.333	16.985	101.000	20.615	98.625	19.992	95.727	15.219	111.750	24.794	1.234	0.312
OACS	244.111	26.722	231.000	17.131	232.500	28.725	223.363	22.944	242.625	40.113	0.941	0.450

† Full scale names are in Table 2

a China (3); Korea (2); Nepal (1); Bangladesh (1); Zimbabwe (1)

### 3.7 Participants’ Performance on the ACS Subscales by Program of Study (N = 45)

Foundation stage students scored significantly higher than their secondary school level counterparts on the ROS variable (Table 10).

**Table 10:** Means, standard deviations and T-values by program of study (N = 45)

Scale†	Secondary (n = 14)		Foundation (n = 31)		ANCOVA	T	P
	Mean	SD	Mean	SD	F	(*df* = 43)	(2-tailed)
PCS	115.428	14.548	118.704	14.949	0.029	-0.687	0.496
ROS	41.285	9.793	48.193	9.988	0.060	-2.160	0.036*
NCS	107.714	17.035	100.907	20.640	1.003	1.068	0.292
OACS	233.071	26.753	234.548	28.233	0.006	-0.165	0.870

* p < .05 (2-tailed)

† Full scale names are in Table 2

### 3.8 Participants’ Performance on the ACS Subscales by Duration of Stay in Australia (N = 45)

No significant differences were obtained on all the subscales (Table 11).

**Table 11:** Means, standard deviations and F-values for length of stay in Australia (N = 45)

Scale†	0 – 1 Month (n=3)		2 – 6 Months (n=14)		7–12 Months (n=27)		13–18 Months (n=1)		F	P
	Mean	SD	Mean	SD	Mean	SD	Mean	SD	(*df*=3;44)	(2-tailed)
PCS	115.000	6.082	122.500	16.681	116.148	14.067	100.000	-	1.118	0.353
ROS	52.333	13.576	50.214	6.941	43.592	10.845	35.000	-	2.179	0.105
NCS	115.333	17.156	104.142	25.527	100.333	16.150	125.000	-	0.992	0.406
OACS	248.000	31.000	242.357	26.057	228.296	27.755	233.000	-	1.086	0.366

† See Table 3 for full scale names

## 4. Discussion

Results of this study showed that the coping strategies most used by international adolescent students were Focus on Solving the Problem, Work Hard to Achieve and Seeking Relaxing Diversions. These findings are consistent with the conclusions of Frydenberg and Lewis (1993b). However, the results of the least used strategies in this study varied differently for the gender comparison (for female students; Seeking Professional Help and Social Action, and for male students; Tension Reduction and Not Coping) and the transitional level experiences (for the ‘Yes’ students; Not Coping and Social Action, and for the ‘No’ students; Ignore the Problem and Tension Reduction).

In relation to findings of the coping styles used by these international adolescent students, Productive coping indicated the style that was mostly utilized and Reference to Others was the least used style. These findings again appeared to be consistent with the results of the findings by Frydenberg and Lewis (1993b) and Frydenberg, Lewis, Kennedy, Ardila, Frindte and Hannoun (2003). Frydenberg et al.’s (2003) study showed that Palestinian adolescent students (mean = 3.54) used this style more often than the Australian (mean = 3.28), Colombian (mean = 3.33) and German (mean = 3.10) subjects.

As reported earlier, the most noticeable difference amongst the coping strategies used by international male adolescent students was Physical Recreation. Again, this finding is consistent with the results of Frydenberg and Lewis (1993b), where in Australian context; boys tend to be more focused on sports than do girls. Similarly, in a Polish study by Guszkowska (2005) where the study examined gender differences of the physical fitness of 253 Polish high school students. Guszkowska concluded that boys who are physically fit seemed to be better equipped to cope with stress and more able to maintain better mood, well-being and health than do girls.

Other factors that should have been examined in this study are international students’ adjustment experiences to Australian educational system, language differences and social issue such as homesickness. In a study by Bell and Bromnick (1998), they investigated how first year university students (late adolescents) in the north of England, cope with the transition of living away from home for the first time. The results of their study indicated the feelings of homesickness were prominent during the first two weeks of moving to university but the feelings reduced over time. In this study however, only one of the 45 respondents (i.e., a male student) recorded ‘homesickness’ as the main concern and nobody reported on ‘culture shock’ issues. We had a short interview discussion with one of the participants (in this study) on this issue and the student gave her/his view on this by saying that if the survey was distributed two months earlier, ‘homesickness’ would be high on the agenda.

The coping strategy; Seeking Professional Help was also indicated in this study as one of the least used strategy by these international students. This part of the findings of this study confirmed the findings from previous studies (Chen, 1996; Constantine et al., 2005; Myburgh et al., 2002; Hashim, 2003; Nasrin, 2001; Zhai, 2002) that reported international students were generally not open to seeking professional help (such as student counseling service) to address their personal or cultural adjustment or academic problems. However, the following statement by Zhai (2002) may provide an explanation for this,

In addition, although student counseling services were designed to serve all students, not all counselors were trained to serve the specific needs of international students. Such problems are not unique and were also encountered by Mundia’s (2012a; 2012b; 2010a; 2010b; 2010c; 2010d; 2010e) studies. Therefore, international students may not trust the counselors and may question their ability to solve their personal problems. Furthermore, because of cultural differences, it is difficult for many international students to open their hearts and share their personal concerns with someone they do not know very well. This might help to explain the reason why the least used coping style was reference to others (ROS subscale in the present study).

Moreover, Wan et al. (1992) emphasized that institutions of higher education must be persistent in order to reach out to these international students so they can take advantage of the intervention and pastoral care programs that have readily been made available to them. The findings of the present study indicated that students who have been away from family used twelve of the 18 coping strategies as compared to students who reported that it was their first time being away from family. Zhai (2002) stated “Students who came to U.S. earlier may have the potential to become effective peer helpers”, (p. 15). This statement may also be true for the students in this study. Students who have been away before can help the students who are first timers by discussing successful methods for coping with cultural, personal and academic differences and difficulties. Having their fellow international students to interact with could help new students feel at ease in the new environment away from the safety and security of the usual habitat (home).

## 5. Conclusion and Recommendation for Future Research

This study was conducted with the hope of identifying the coping behaviors of international adolescent students while studying in Australian institutions. The three most used coping strategies employed by these students belonged to the productive coping group. The overall findings of this study indicated that these international adolescent students were coping well with their stresses in Australia. However, these international students should be encouraged to interact with the local (Australian) students, because by interacting, their language and communication skills would improve and this would also provide opportunities for them to understand and adjust to the Australian culture. Exposing these international adolescent students to the early problems of academic or adjusting issues will prepare them to cope even better when they go to universities in Australia or other parts of the world.

The present study specifically targeted the international students in the late adolescence age group. It is recommended that this study be replicated to cover a larger sample of international adolescent students to gain additional insights. Furthermore, a study of this nature welcomes other instrumentation and mixed methods to be used in order to explore other factors related to international adolescent students’ coping behavior. Above all, we recommend that those students who are identified to have difficulties should be encouraged to undergo voluntary counseling to relieve the distress.

## 6. Limitations of the Study

The present study has three main limitations. First, the sample of this study was considered small for a country that has thousands of international adolescent students. Being a small exploratory study, the findings cannot therefore be generalized to all international adolescent students studying in Australia. Second, a qualitative interview component is missing but was essential and needed to complement or supplement data from the quantitative survey. Such triangulation of methods and data was considered important in view of the fact that we compared findings from the same participants by gender, educational level, country of origin, age-groups, and so on. It is vital to note that international students from different cultural backgrounds have both differences and similarities in terms of their coping behavior. In addition, age-related differences (e.g. early, middle, and late adolescents) in coping have contrasting outcomes in terms of the coping strategies used by these groups of adolescents. For instance, Frydenberg and Lewis (2000) indicated the most significant age-related findings by saying,

… older adolescents are more likely to blame themselves for their stresses and to resort to tension reducing strategies such as eating or drinking. Older adolescents are also less likely to work hard, seek professional advice, or utilize spiritual support. (p. 741)

Third, we used only one instrument (the ACS) to collect the data for the study. Though appropriate for the purpose, the concurrent or criterion-related validity of this scale was supposed to have been obtained by correlating its subscales with subscales of other similar instruments that assess coping strategies and styles in adolescents. The instruments used in previous studies were mainly for adult international students (e.g. the Study Abroad Stress Survey, SASS and the Coping Skills Inventory, CSI) used by Hashim (2003). Specific instruments for assessing adolescent coping in international settings or foreign countries need to be either identified or developed and used in future research.
